# Bird diversity along an urban to rural gradient in large tropical cities peaks in mid-level urbanization

**DOI:** 10.7717/peerj.16098

**Published:** 2023-10-09

**Authors:** Marcela Suarez-Rubio, Paul J.J. Bates, Thein Aung, Nay Myo Hlaing, Sai Sein Lin Oo, Yu Kay Zin Htun, Saw Myat Ohn Mar, Aye Myint, Thin Lae Lae Wai, Pann Mo Mo, Lutz Fehrmann, Nils Nölke, Christoph Kleinn, Swen C. Renner

**Affiliations:** 1 Institute of Zoology, Department of Integrative Biology and Biodiversity Research, University of Natural Resources and Life Science, Vienna, Austria; 2 Harrison Institute, Sevenoaks, UK; 3 Myanmar Bird and Nature Society, Yangon, Myanmar; 4 Mandalay University, Mandalay, Myanmar; 5 Myeik University, Myeik, Myanmar; 6 Forest Inventory and Remote Sensing, University of Göttingen, Göttingen, Germany; 7 Ornithology, Natural History Museum Vienna, Vienna, Austria

**Keywords:** Bird community composition, Species richness, Urbanisation, Shift in abundance, Land-cover change, Global change, Species diversity

## Abstract

The gradient from natural to urban areas strongly associates with the structure of avian communities over that gradient. Most research on urban birds is from temperate areas and knowledge from tropical Southeast Asia is lacking. We examined bird species diversity, relative abundance, and species composition along an urban to rural gradient in three Myanmar cities, and assessed potential environmental factors responsible for the changes. We counted birds within 40 point-count sites with 50-m fixed-radius in three large cities of Myanmar, namely Mandalay, Mawlamyine, and Myeik. We distinguished four urban habitat types (Downtown–urban, University Campus–suburban, Paddy Field–agriculture, Hill–forest). We classified all species into migrant or resident and into major feeding groups and related with several environmental parameters such as ‘impervious surface’. We counted 5,423 individuals of 103 species with roughly equal species diversity between the three cities. Rock Pigeon (*Columba livia*) was the most frequent species. The species composition differed significantly between the four major habitat types. Omnivores were more abundant in the city center than all other functional groups. Interestingly, insectivores were also predominant in the city center. In addition, more generalist’ species occurred towards the city center compared to the periphery, indicating that the periphery has increased relevance for specialized birds. We found some marked differences in species composition between the three cities of Mandalay, Mawlamyine, and Myeik. Additionally to species composition, species diversity and relative abundance differed significantly between each of the four major habitat types in all three cities.

## Introduction

Urbanization is one of the principal processes of modifying habitats since industrialization started and is one of the major causes of species extinction (Czech, Krausman & Devers, 2000; Marzluff, 2001; Sol et al., 2014). The continuous growth of human populations (United Nations Department of Economic and Social Affairs Population Division, 2022) has promoted urban expansion which in some regions is even larger than population growth (Mahtta et al., 2022). Natural environments are being replaced by urban areas (Seto et al., 2011). Urban areas are characterized by fragmented and disturbed environments, high densities of artificial structures and hard surfaces (*e.g.*, rooftops, driveways, parking lots) and few isolated natural habitats (Cadenasso, Pickett & Schwarz, 2007). Thus, urbanization results in changes in land cover, hydrological systems, biogeochemical cycles, climate, and biodiversity (Grimm et al., 2008). Indeed, their ecological impact goes beyond city limits, generating environmental changes at local and even global scales (Aronson et al., 2014). As a consequence, many organisms, particularly birds, are highly affected by urbanization (*e.g.*, Beissinger & Osborne, 1982; Biamonte et al., 2011; Crooks, Suarez & Bolger, 2004; Reale & Blair, 2005; Sorace & Gustin, 2010; Suarez-Rubio, Leimgruber & Renner, 2011).

In general, bird diversity is reduced while abundance and density increase, in urban as compared to low density rural areas (Chace & Walsh, 2006; McKinney, 2008; Suarez-Rubio, Leimgruber & Renner, 2011). It varies by geographic regions (Saari et al., 2016), depend on factors such as density of urbanization, the extent of hard surfaces, and the distribution of green spaces (Aronson et al., 2014). In general, one may assume that urbanization changes bird species composition as a function of urban development (Suarez-Rubio et al., 2016). Urban and rural species communities are often similar in species diversity, but can differ significantly in species composition, indicating that urbanization is a major cause of biotic homogenization (Clergeau et al., 2006; Filloy, Grosso & Bellocq, 2015; McKinney, 2006; Morelli et al., 2016). Urban bird communities are usually dominated by a few, sometimes introduced species, with granivores, omnivores, and cavity-nesting species being particularly favored (Beissinger & Osborne, 1982; Blair, 1996; Fernández-Juricic, 2001; Ortega-Alvarez & MacGregor-Fors, 2009). These patterns have been observed for forest, desert scrub, and grassland habitats (Chace & Walsh, 2006). However, studies outside temperate areas are still underrepresented (Ortega-Álvarez & MacGregor-Fors, 2011) and whether birds respond similarly in urban tropical areas still requires further assessment. Nonetheless, diversity patterns and composition of urban bird communities are affected by abiotic factors and ecological and evolutionary processes, *e.g.*, species interactions, dispersal, and natural selection (Alberti et al., 2020; Faeth, Bang & Saari, 2011; Marzluff, 2012), with urbanization potentially altering patterns and processes.

Although Southeast Asia is experiencing rapid human population growth, the level of urbanization is still fairly low compared to other regions (Jones, 2013). However, urban areas have been rapidly increasing in the last decades (Jones, 2013). In 2010, around 42% of Southeast Asia’s population lived in urban areas, which is twice the proportion compared to 1970 (Jones, 2013). By 2030, it is projected that this figure will increase to 56% (Hugo, 2019). Nevertheless, information on the relationships between urbanization and avian communities in Southeast Asia and particularly in Myanmar is scarce (Chace & Walsh, 2006; Magle et al., 2012; Muriel et al., 2021; Suarez-Rubio et al., 2016). Myanmar is one of the world’s most biodiversity-rich countries (Bates et al., 2021; Mittermeier et al., 2011; Sodhi et al., 2004) and has a relatively high bird diversity in the forested areas (Kyaw et al., 2021; Renner & Bates, 2020; Renner et al., 2015; Oo et al., 2019; Oo et al., 2022; Oo et al., 2020; Oo, Kham & Renner, 2019). However, little is known about the effects of urbanization on Myanmar’s avifauna in large cities compared to rural sites (Päckert et al., 2019; Renner & Bates, 2020; Suarez-Rubio et al., 2020).

We analyzed bird species diversity and relative abundance along an urban to rural gradient in and around Mandalay, Mawlamyine, and Myeik, three large and locally important cities. We also assessed environmental factors that could potentially explain the changes in the bird community composition. We hypothesized: (1) Species diversity will decrease with increased human population density and increased proportion of hard surfaces, while relative abundance will increase with increasing urbanization. (2) Species composition will shift along the urban to rural gradient from specialists in rural areas to generalists in urban areas. Consequently, we expected at the city center (Downtown—urban) few generalist species with high abundance (*i.e.,* omnivore species); outside the city centers (*i.e.,* University Campus—suburban) and in agricultural/forested areas (Paddy Field—agriculture; Hill—forest) more specialized species. (3) Functional groups should vary along the urban to rural gradient with granivores and omnivores becoming dominant towards the urban downtown and decrease towards the periphery.

## Material and Methods

**Figure 1:**
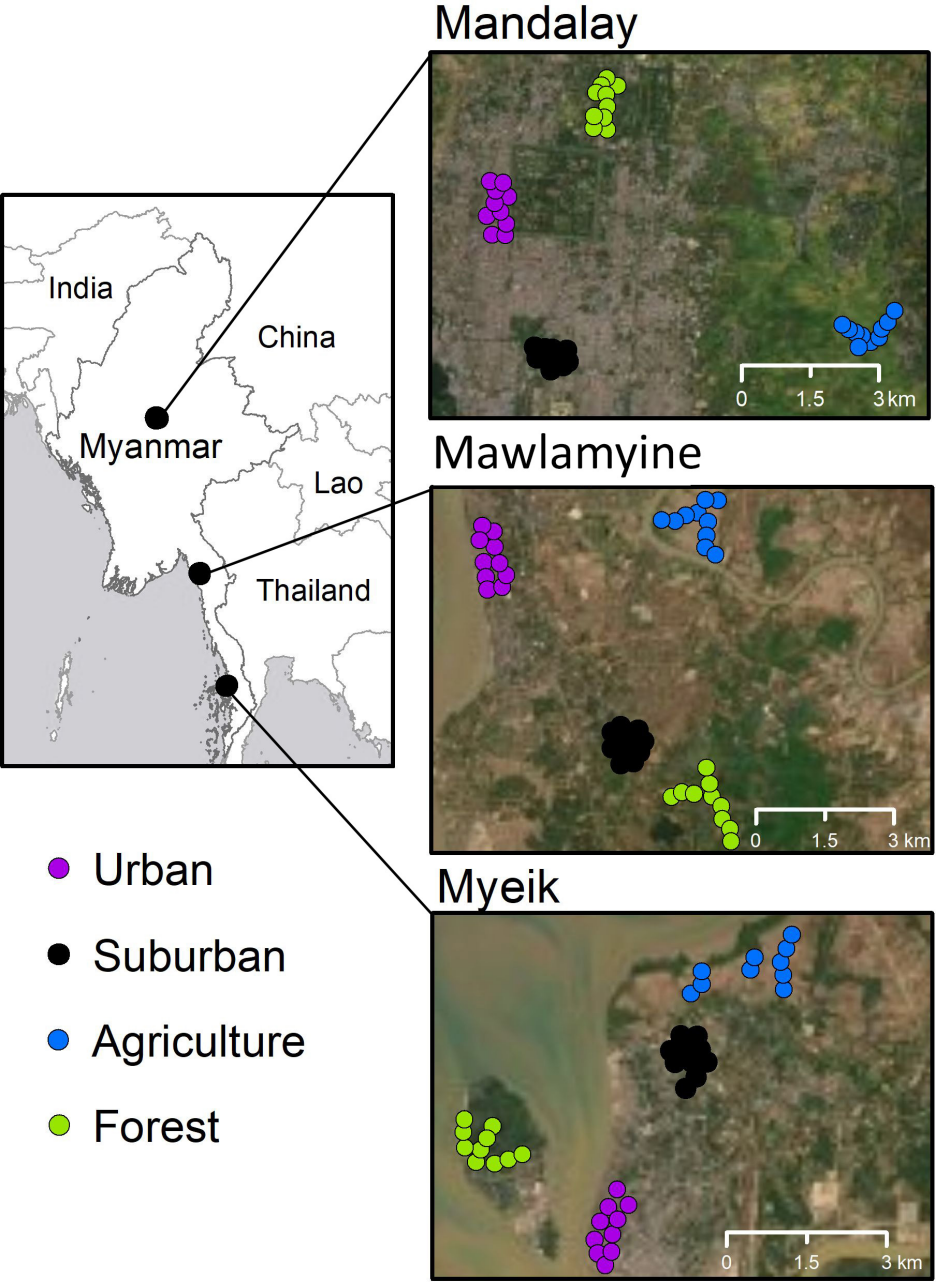
Study areas (overview on the left) in Myanmar and locations of point counts (colored circles) in Mandalay, Mawlamyine, and Myeik. Each circle represents a point count site in one of the four habitat types in each city. The Aerial photos are available from the base map of ArcGIS v10.6.1.

We surveyed birds in three large cities of Myanmar (Fig. 1): Mandalay (164 km^2^ urban area; 1,726,889 inhabitants; 75 m a.s.l.; 11–41 °C; 50 cm annual mean precipitation; GDA, 2015), Mawlamyine (219 km^2^ township area; 254,661 inhabitants; 6 m a.s.l.; 15–40 °C; 626 cm; GDA, 2020), and Myeik (8 km^2^ urban area; 52,000 inhabitants in Myeik township; 25 m a.s.l.; 22–38 °C; 410 cm; GDA, 2019). We selected four habitat classes, which were based on the criteria of Suarez-Rubio et al. (2016) and were distinguished through expert interpretation of high-resolution remote sensing imagery. For each city, ten point count sites (point ID) were placed in each of the four habitat classes, namely: (1) ‘Downtown’, the area of the city center with the highest human population density (urban); (2) ‘University Campus’ which was a park-like structure (suburban) in all three cities, typically outside the urban core; (3) ‘Paddy Field’ which was a rural-agricultural area on the margin of the cities (agriculture) with low human population and characterized by rice paddy, irrigation channels, and access paths; and (4) ‘Hill’, which was a hill site relatively close to each city center, covered with bush, shrub and forest. Mandalay Hill is a forested area isolated by agriculture and settlements; Mawlamyine Hill is part of a larger isolated forest area with no connection to natural forests; and Myeik Hill is a forested area located on an island about 500 m offshore from the city center.

We recorded all birds within a 50-m-fixed-radius at each point count site. We counted for 10 min as a group with the same core observer-team (M.S.R., T.A., accompanied by a varying number of co-authors and students) throughout all 120 point IDs. We employed the same method as Suarez-Rubio et al. (2016), to allow for comparison of datasets. By using 10-minute intervals, we minimized the probability of double counting and maximized the probability of recording all species at any given sampling point (Bibby, 2000; Suarez-Rubio et al., 2016). We registered all birds recognized as an individual and assessed the total number of all individuals heard or seen within each point count site. Surveys were done from just before sunrise to 9 a.m. (Suarez-Rubio et al., 2016), in one instance until 10:20 a.m. local Myanmar time. We surveyed for four consecutive days in each city: Mandalay 23–26 June 2019; Mawlamyine 9–12 March 2019; and Myeik 3–6 March 2019.

We kept a minimum distance of 250 m in-between point count sites to reduce spatial auto-correlation and considerably decrease the likelihood of counting the same bird twice (Suarez-Rubio et al., 2016). In the ‘Hill’ sites, we selected points along the main road towards the top/pagoda and used stairways to minimize interference with religious activities (Suarez-Rubio et al., 2016). In the other three habitat types, we kept on roads or walking paths to have similar observational conditions and reduce bias by diversification of micro-habitats (Suarez-Rubio et al., 2016). The characteristics of habitat and general structure within each of the four habitat types and between the three cities are homogenous and follow Suarez-Rubio et al. (2016).

We excluded several counted individuals *a priori* from the analysis since no safe identification was possible: one harrier (Myeik), one pipit (Mawlamyine), eight warblers (Mawlamyine, three; Myeik, five), one swift (Mawlamyine), and one oriole (Mawlamyine). We merged Western Cattle Egret and Eastern Cattle Egret into ‘Cattle Egret’, even if partially the two taxa could be distinguished by plumage, which was, however, not possible in Mandalay during our study time. We initially used ‘Jungle crow’ in some instances but we later merged these with Large-billed Crow.

We classified all species into migrant or resident but disregarded any finer distinctions such as partial migrant or seasonal/altitudinal migrant. We also categorized each species for its major feeding group: carnivore (feeding on mainly vertebrate prey); granivore (feeding on seeds and other plant parts); herbivore; insectivore (feeding on arthropods); nectarivore (including frugivores); omnivore; and piscivore (feeding mainly on submerged animals including fish and macro-zoo-benthos). These feeding groups represented the main diet during reproductive periods per species. Both classifications are kept intentionally simple and might not include all nuances of migration status or diet for each species.

### Environmental variables, land cover, and NDVI

At each of the bird counting sites we established a fixed-area, circular-sample plot for detailed assessments of tree resources. A plot radius of 15 m was used in areas with relatively high tree density (*e.g.*, gardens in the city center and dense tree cover inside forest), while larger plots of 30 m radius were used in open areas with sparse tree cover (*e.g.*, on the Paddy Fields/agriculture). For each selected tree we measured diameter at breast height (DBH, measured at 1.3 m height), tree height, direction angle and horizontal distance to the plot center. Because a direct species identification of all trees was not possible, we assigned classes to distinguish trees and palms and separated deciduous and coniferous trees.

In addition to tree resources, we assessed the dominant land cover class of the plot (urban, pasture, cropland, shrubland, forest) and identified relevant habitat elements (large fig or other special habitat trees, tree/shrub groups, forest patches, open water surface, *etc*.) in the surrounding of the plot. At each location we took a spherical 360° photo using a Ricoh Theta camera for documentation. These 360° pictures were converted into binary images to determine a gap fraction (using ImageJ) and to separate sky from non-sky pixels. This information was used as an indicator for ‘visibility’ and therefore expected to have an influence on bird counts.

### Remote sensing analysis

For all three cites, we acquired the most recent cloud-free Sentinel 2 satellite image (L1-C, orthorectified Top-Of-Atmosphere reflectance) and calculated the Normalized Difference Vegetation Index (NDVI). NDVI is known to be positively correlated to the amount of green vegetation. The pixelwise NDVI was aggregated (mean and standard deviation) over different radii of 50 m (*i.e.,* covering the area of bird observations), 75 m, 100 m and 125 m around each bird point count site. In addition, we determined the proportion of open water surface inside these different radii to account for and normalize plots close to the waterfront or in wetlands. Mean NDVI was then re-scaled to mean NDVI per land surface area. We measured the morning temperature at the beginning of the bird surveys, typically at 5:30 am.

### Statistical analyses

To characterized the bird communities, we standardized the samples based on sample completeness (*i.e.,* coverage) (Roswell, Dushoff & Winfree, 2021). Coverage is a measure that describes how well a sample captures the diversity of the whole community, including species that have not yet been detected (Roswell, Dushoff & Winfree, 2021). It estimates the proportion of individuals in the (whole) community that belong to species present in the sample (Roswell, Dushoff & Winfree, 2021). By standardizing samples by coverage, we were able to compare data among the three cities and reduce bias in biodiversity comparisons (Roswell, Dushoff & Winfree, 2021). Once the samples were standardized, we calculated Hill diversity (Chao et al., 2014; Roswell, Dushoff & Winfree, 2021). Hill numbers are a mathematically unified family of diversity indexes that incorporates species richness and relative abundance (Chao et al., 2014) and allows to explicitly choose how sensitive the diversity metric is to rare species. We used a Hill number with *q* = 1, *i.e.,* Hill Shannon diversity, which emphasizes neither rare nor common species to allow characterizing gradients in biodiversity (Roswell, Dushoff & Winfree, 2021). Sample robustness was measured with sample-size-based diversity accumulation curves, in which the expected Hill Shannon diversity is plotted as a function of sample size using the number of individuals (Chao et al., 2014). The use of number of individuals is considered the best measure of sampling effort (Willot, 2001) given that this allows to make a direct comparison of species diversity among the three cities. We also used coverage-based diversity curves to plot expected diversity as a function of interpolated (observed) and extrapolated (expected) coverage. These analyses were performed using the R package iNext (Hsieh, Ma & Chao, 2022).

To test the effect of city and habitat types (and their interaction) on Hill Shannon diversity and relative abundance, we performed two separated Generalized Least Squares (GLS) models including the variance structure (Zuur et al., 2009). GLS models extend the ordinary least squares estimation of the normal linear model by providing the possibility of unequal error variances. We tested and selected the best variance structure for each model. In both models, city, habitat and pairwise interaction were used as the explanatory variables. The Hill Shannon diversity or relative abundance (number of individuals counted per species per point ID) was used as the response variable. GLS models were generated using the R package nlme (Pinheiro et al., 2021) and the figures were prepared with ggeffects (Lüdecke, 2018).

To assess differences in bird community assemblages among habitat types and cities, we performed a non-metric multidimensional scaling (NMDS) using Bray-Curtis distance (McCune & Grace, 2002). We searched for outliers between bird species and habitat types and cities, using Bray-Curtis distance with a cut-off of two standard deviations (McCune & Grace, 2002). We ran the NMDS using Bray-Curtis distance, with a first approximation run of 6D stepping down to 1D solution, starting 500 runs from a random configuration and 500 iterations. We selected 3D as the final solution, using the starting configuration that worked best, and one real-run (McCune & Grace, 2002). We used a multi-response permutation procedure (MRPP) using Bray-Curtis distance to determine statistical differences among the groups formed in the NMDS for habitat types and cities. The outlier analysis, NMDS, and MRPP were performed using PC-ORD 6 for Windows (McCune & Mefford, 2011).

To identify the most important environmental factors out of a set of variables characterizing urbanization and vegetation, first we tested all explanatory variables for collinearity with vif() in the R package usdm (Naimi et al., 2014) and consequently excluded all variables with a VIF > 3 to reduce multi-collinearity. This resulted in reducing the NDVI dataset, in which only the 50 m buffer parameters remained. NDVI min and max values were also highly correlated and those were excluded. This approach retained the parameters city, habitat type, migrant, functional group, land use, terrain, gap fraction, tree basal area, tree density, NDVI land cover 50 m, water proportion 50 m, and impervious surface 50 m. These variables were subsequently used in Generalized Linear Models (GLMs) following the general structure ‘relative abundance ∼ city + habitat type + Migrant + functional group + land use + terrain + gap fraction + tree basal area + tree density + NDVI land cover 50 + water proportion 50 + impervious surface 50’. We then selected the best fitting model out of this set of models based on AICc values to find the most parsimonious model—while we report models within the 95% CI of the model averaging in Table 1, we omitted any model with uninformative parameters as discussed by Arnold (2010). Since our sample size was relatively small, we based our selection procedure on AICc. We scaled bird relative abundance data for the GLMs to avoid problems of parameters with highly different scales (scale() in R). We performed all lmer() with the package lme4 and model averaging following Burnham & Anderson (2002) in the package AICcmodavg in R (R Core Team, 2018).

**Table 1:** Model averaging results of relative abundance of birds in three major cities of Myanmar retaining the three best-fitted models.

Model	K	AICc	ΔAICc	AICc weighted	Cumulative Weight	Restricted log-likelihood	Pseudo -R^2^	Predictors in model
ma13	14	2665.02	0.00	0.46	0.46	−1318.29	0.09	Habitat type, functional group, NDVI land50, water50, impervious surface50
ma15	12	2666.09	1.07	0.27	0.72	−1320.88	0.08	Habitat type, functional group, water50
ma14	13	2668.11	3.09	0.10	0.82	−1320.86	0.08	Habitat type, functional group, water50, impervious surface50
ma12	16	2668.25	3.23	0.09	0.91	−1317.83	0.09	City, habitat type, functional group, NDVI land50, water50, impervious surface50
ma7	18	2670.60	5.58	0.03	0.94	−1316.93	0.09	City, habitat type, migrant, functional group, gap fraction, tree density, water50, impervious surface50
ma17	16	2671.48	6.46	0.02	0.95	−1319.45	0.08	Functional group, land use, NDVI land50, water50
ma11c	18	2672.39	7.37	0.01	0.97	−1317.83	0.09	City, habitat type, functional group, tree basal area, tree density, NDVI land50, water50, impervious surface50
ma16	17	2672.50	7.48	0.01	0.98	−1318.92	0.08	Functional group, land use, NDVI land50, water50, impervious surface50
ma4	17	2672.50	7.48	0.01	0.99	−1318.92	0.08	Functional group, land use, NDVI land50, water50, impervious surface50
ma6	20	2672.79	7.77	0.01	1.00	−1315.94	0.09	City, habitat type, migrant, functional group, gap fraction, tree basal area, tree density, NDVI land50, water50, impervious surface50
ma18	15	2675.58	10.56	0.00	1.00	−1322.54	0.08	Functional group, land use, water50
ma3	22	2678.26	13.24	0.00	1.00	−1316.59	0.09	City, habitat type, migrant, functional group, land use, NDVI land50, water50, impervious surface50
ma10	28	2688.79	23.77	0.00	1.00	−1315.52	0.09	City, habitat type, migrant, functional group, terrain, gap fraction, tree basal area, tree density, NDVI land50, water50, impervious surface50
ma5	31	2696.00	30.98	0.00	1.00	−1315.92	0.09	City, habitat type, migrant, functional group, land use, terrain, gap fraction, tree basal area, tree density, water50, impervious surface50
ma2	32	2696.33	31.31	0.00	1.00	−1315.02	0.09	City, habitat type, migrant, functional group, land use, terrain, gap fraction, tree basal area, tree density, NDVI land50, water50, impervious surface50
ma1	7	2706.84	41.82	0.00	1.00	−1346.36	0.07	Habitat type, city
ma8	6	2714.22	49.21	0.00	1.00	−1351.07	0.06	Tree density, NDVI land50, water50, impervious surface50

**Notes.**

Pseudo-R^2^ calculated as McFadden’s R^2^.

## Results

There was a total of 5,423 individual bird records of 103 species. Additional 12 observations in six taxa could not be identified to species and these 12 observations were omitted. In Mandalay, we recorded 2,688 individuals in 57 species; in Mawlamyine, 1,094 individuals in 52 species; and in Myeik, 1,641 individuals in 59 species. While the most abundant bird was the Rock Pigeon (*Columba livia*) in all three cities, the top five most abundant species differed between the cities. In Mandalay, Rock Pigeon accounted for almost a quarter of all counts (1,245; probably because of active feeding in the then touristic areas of Mandalay) and was seven times higher than the second most counted species, the House Sparrow with 173, followed by Himalayan Swiftlet with 145, Glossy Ibis with 134, and House Swift with 121. In Mawlamyine, we recorded Rock Pigeon 279 times, House Crow 187 times, Eurasian Tree Sparrow 120 times, Asian Palm Swift 76 times, Common Myna 75 times. In Myeik we found the Rock Pigeon 413 times, Edible-nest Swiftlet 241 times, White-rumped Munia 153 times, Scaly-breasted Munia 142 times, and House Crow 126 times (Table S1).

**Figure 2:**
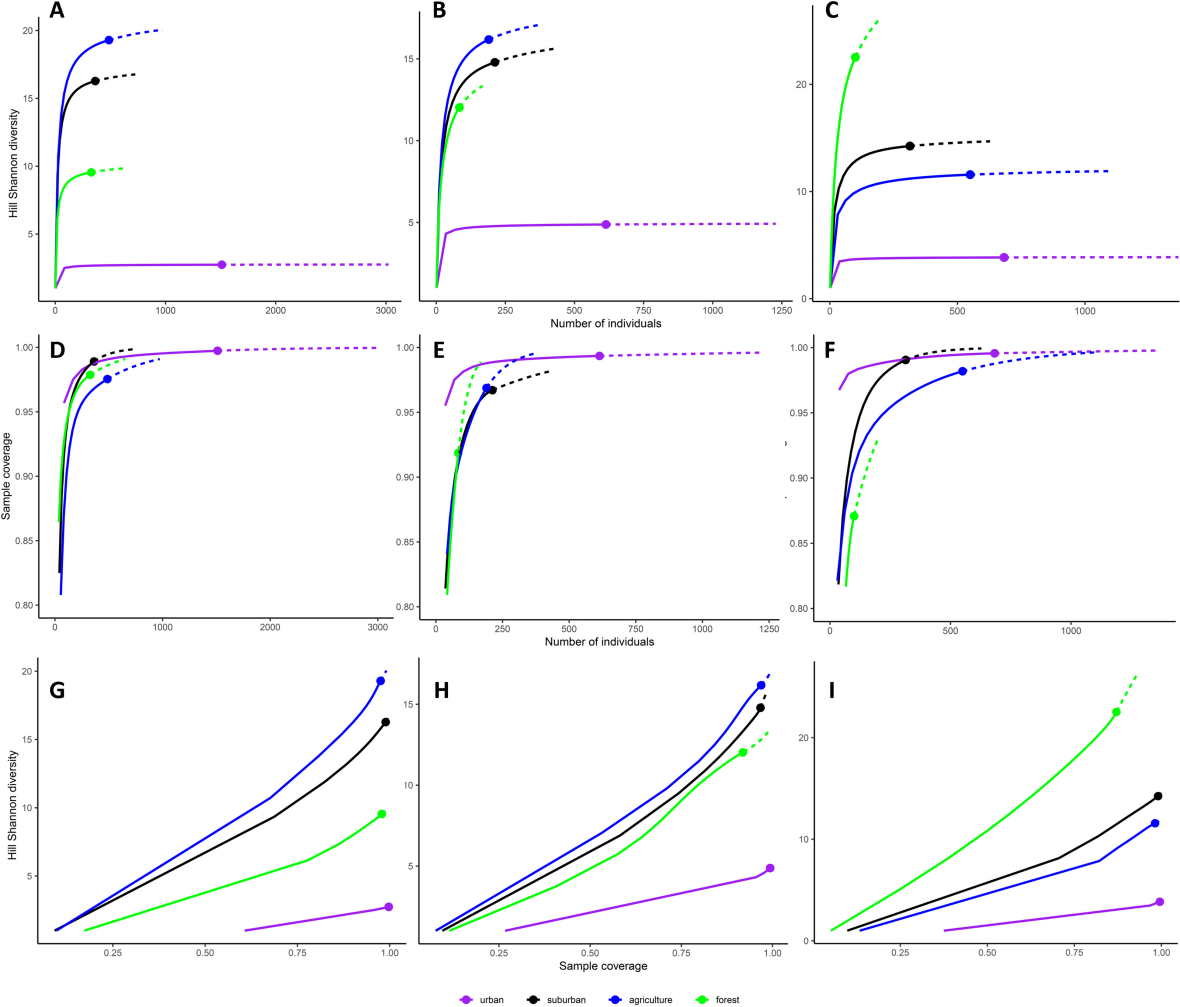
Diversity accumulation curves for each habitat type per city. Hill Shannon diversity as a function of number of individuals (*i.e.,* sample-sized-based; A–C), sample coverage for rarefied samples as a function of number of individuals (D–F), and Hill Shannon diversity as a function of sample coverage (*i.e.,* coverage-based (G–I) for Mandalay (A, D, G), Mawlamyine (B, E, H) and Myeik (C, F, I)). Continuous lines represent interpolation (*i.e.* observed) and dashed lines represent extrapolation (*i.e.* expected) of bird species diversity. Color coding of habitat types is identical as for Fig. 1 (map).

Diversity accumulation curves were saturated for the urban habitat type in all three cities (Figs. 2A to 2C) and for suburban and agriculture in Myeik (Fig. 2C). They were close to saturation for suburban and agriculture in Mandalay (Fig. 2A) and Mawlamyine (Fig. 2B); whereas for forest, diversity did not reach saturation in Mawlamyine (Fig. 2B) and Myeik (Fig. 2C). Sample coverage reached an asymptote with the sample effort employed in all habitat types in Mandalay (Fig. 2D), Mawlamyine except for forest and agriculture (Fig. 2E) and Myeik except for forest (Fig. 2F). Sample coverage on Hill Shannon diversity was on average 99.4% for urban in all three cities and suburban in Myeik; 97.7% for agriculture in all three cities, suburban in Mandalay and Mawlamyine, and forest in Mandalay; and 89.5% for forest in Mawlamyine and Myeik (Figs. 2G to 2I).

**Figure 3:**
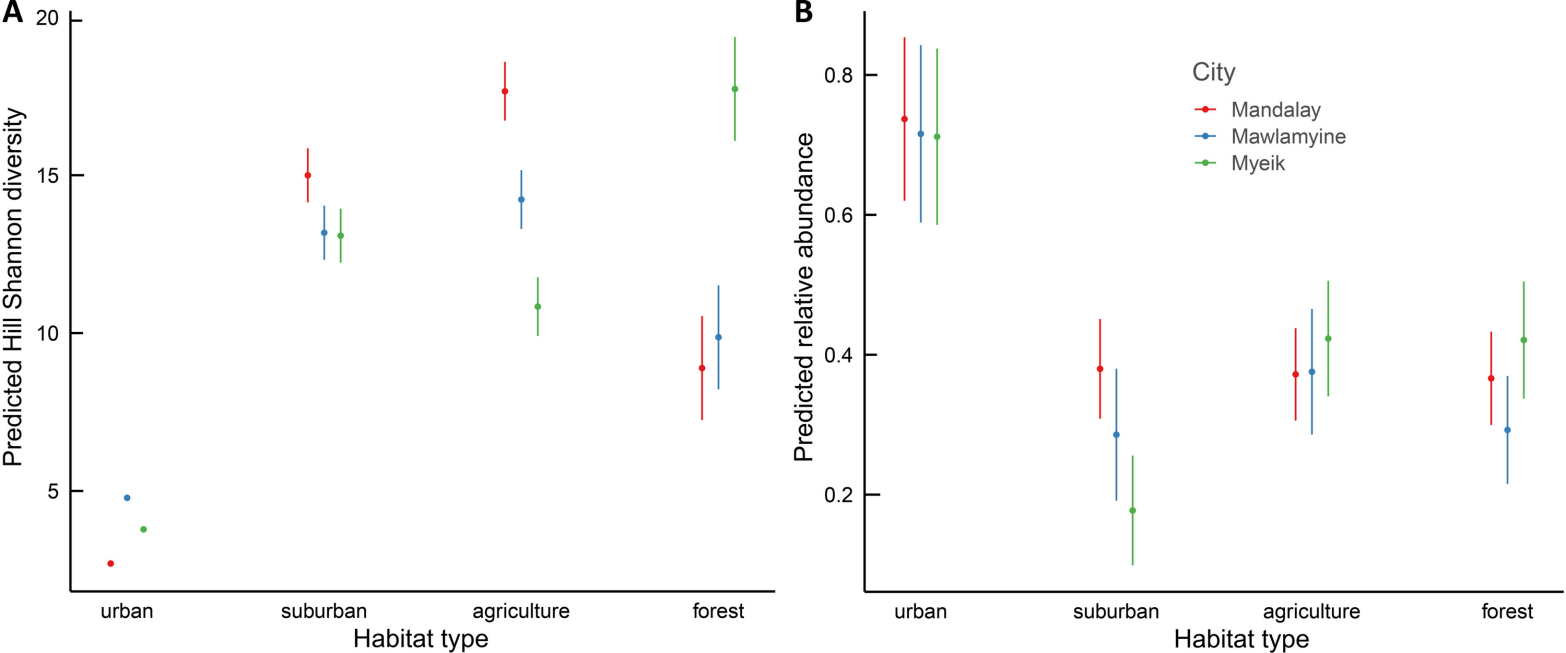
Generalized Least Squares models results for Hill Shannon diversity (A) and relative abundance (B) along the urban to rural gradient in three major cities in Myanmar. The filled circle indicates the mean and the bar 95% confident intervals.

Hill Shannon diversity differed between the three cities (*F*_2, 216_ = 1810.32, *p* < 0.001), the four habitat types (*F*_3, 216_ = 1117.57, *p* < 0.001) and their interaction (*F*_6, 216_ = 42.95, *p* < 0.001). Agriculture and suburban had the highest Hill Shannon diversity and urban had the lowest, following a hump-shaped curve in Mandalay and Mawlamyine (Fig. 3A). Forest had low diversity compared with suburban and agriculture except in Myeik, in which forest had the highest diversity. Forest diversity in Mawlamyine and Myeik were similarly low. Suburban in Mandalay had a higher diversity compared with suburban Mawlamyine and suburban Myeik. Agriculture in the three cities varied with Mandalay having high diversity (similar to forest Myeik) and Myeik the lowest. In Mawlamyine, diversity in suburban and agriculture were similar and comparable to the diversity in suburban Myeik (Fig. 3A). Relative abundance was similar between the three cities (*F*_2, 1073_ = 0.984, *p* = 0.37), but differed between the four habitat types (*F*_3, 1073_ = 35.559, *p* < 0.001) and there was a significant interaction (*F*_6, 1073_ = 2.824, *p* = 0.009). In all three cities relative abundance was higher in urban downtown compared with the other habitat types (Fig. 3B).

**Figure 4:**
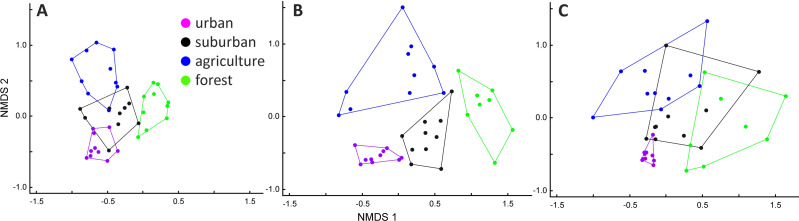
Non-metric multidimensional scaling (NMDS) ordination for Mandalay (A), Mawlamyine (B), and Myeik (C) in Myanmar.

In the analyses of species composition, we identified two sites in Myeik and Mawlamyine in the forest and two species, namely Spotted Dove and Black Drongo, as outliers. The NMDS produced a final optimum 3D ordination space, which represented 68% of the variance in the original species data (NMDS1 = 0.21, NMDS2 = 0.17, NMDS = 0.30, stress = 0.14). The NMDS ordination showed that species composition in the three cities largely overlap, particularly in Mawlamyine and Myeik, less in Mandalay (Fig. S1). However, if considering species composition in relation to habitat types for each city separately (Fig. 4), the four habitat types were clearly separated only for Mawlamyine and there was a slight overlap for Mandalay. Cities and habitat type were different based on the MRPP as well (cities: T = −31.48, *A* = 0.25, *p* ≤ 0.001; habitat: T = −30.35, *A* = 0.29, *p* ≤ 0.001). Species composition in forests was in all three cities clearly distinct from urban and agriculture (Fig. 4), which is likely driven by the island character (real forest island in Myeik, forest patches with sharp edges otherwise). Forest bird composition in Myeik was partially overlapping with suburban, which might be a result of relative proximity to the coast of all sites in Myeik.

Testing how environmental variables relate to bird relative abundance within a 50 m-buffer (all other areas excluded), the best fitted model based on AICc and ΔAICc retained habitat type, functional group, NDVI within 50 m, water within 50 m, and impervious surface within 50 m (model “ma13” in Table 1), followed by a model, including habitat type, functional group, water within 50 m (“ma15” in Table 1). Therefore, the relative abundance of bird species is driven by the amount of green (NDVI), as well as, the impervious surface. In addition, the habitat type along the urban to rural gradient (*i.e.,* urban, suburban, agriculture, or forest) and functional groups of the birds are some important parameters that explain relative abundance. Excluding any of these decreased model-fit considerably.

**Figure 5:**
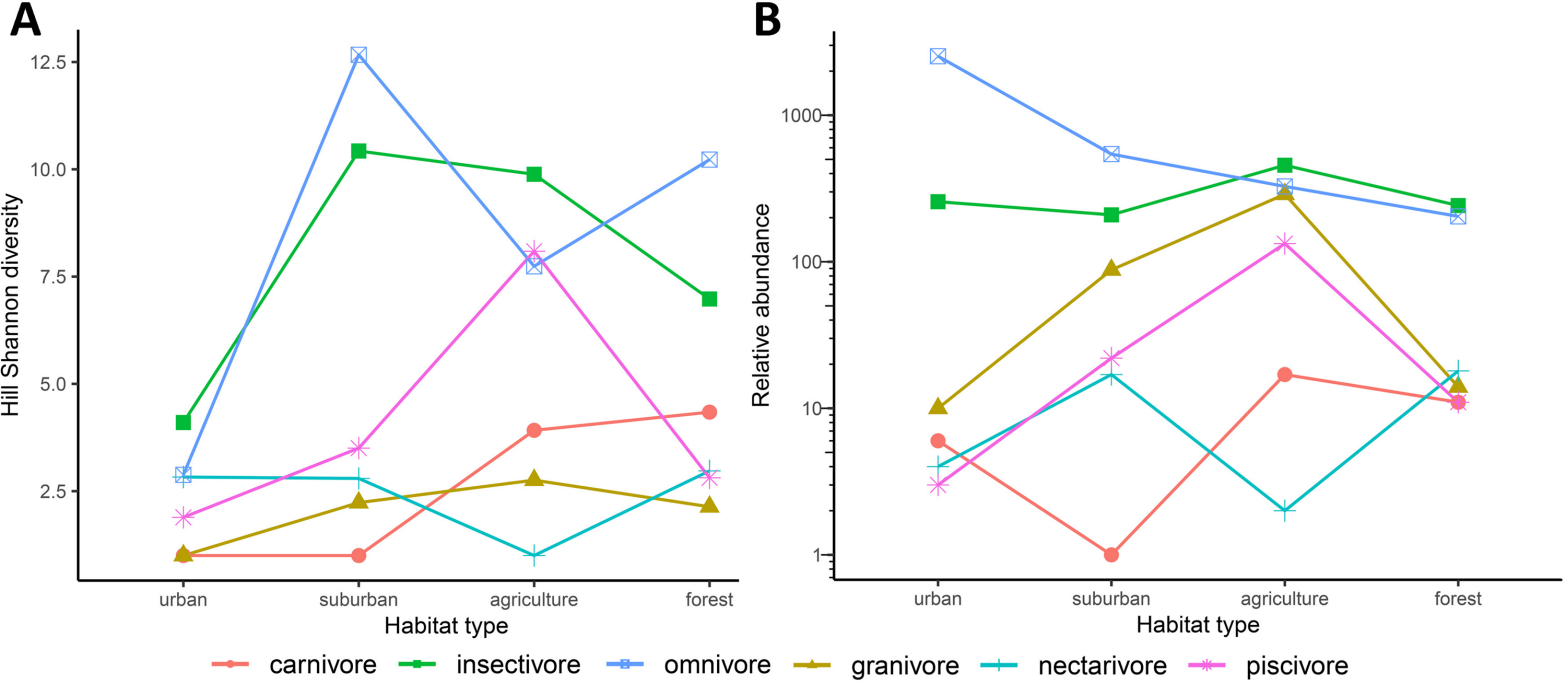
Compartmentation of functional groups along the urban to rural gradient (habitat types) in three major cities (Mandalay, Mawlamyine, and Myeik) in Myanmar. Hill Shannon diversity (A) and mean relative abundance (B) per functional group and habitat type with the three cities merged. Herbivores are omitted (1 record only).

When considering functional groups, some patterns in Hill Shannon diversity and relative abundance occured along the urban to rural gradient. Some functional groups along the urban to rural gradient followed hump-shaped curves in diversity (except for carnivores, omnivores and nectarivores; Fig. 5A), in which few species were found at the two extremes of the gradient. Functional groups for mean relative abundance, however, showed dissimilar patterns. The relative abundance decreased from urban to rural for omnivores, increased along the gradient but then decreased for granivores and piscivores, or had a zigzag pattern for carnivores and nectivores (Fig. 5B). We found omnivores to be more abundant in urban Downtown than all other functional groups, and a large proportion of piscivore, insectivores, granivores and carnivores were found in the mid of the gradient, peaking in agriculture (Fig. 5B). In Myeik, additionally the forest hill site had increased abundance in piscivore bird species (Fig. S2F). The latter might be driven by the location of the forest on an island close to Myeik, which may have increased marine species compared to the other two forest sites in Mandalay and Mawlamyine. The agricultural areas in our study harbored more insectivores than the other habitat types (Fig. 5B). Nectarivores and carnivores were relatively low in abundance in general compared to all other functional groups, and peak in suburban (nectarivores) and agriculture (carnivores) in all instances.

## Discussion

Species diversity and species composition varied between the cities and habitat types, while relative abundance was similar between cities, but not between habitat types, although there was a significant interaction between city and habitat type. Species diversity, relative abundance and species composition differed significantly between each of the four major habitat types within each city *i.e.,* along the urban to rural gradient. But, besides NDVI or impervious surface 50 m, we could not relate any changes to other parameters assessed such as tree resources (Table 1). Our findings support previous research concerning the effects of urbanization on birds. We partially confirmed our first hypotheses that species diversity decreased with increasing human population density (habitat type was used as a proxy). This was the case for Myeik, but for Mandalay and Mawlamyine forest had less species diversity than suburban/agriculture areas, but higher than in urban.

The high species diversity in agriculture (paddy fields), which elsewhere are usually associated with fewer bird species compared to other habitats, such as forested sites (O’Connell, Jackson & Brooks, 2000) could be related to the close proximity of paddy fields to many bushes, trees, and hedge rows in the three cities of Myanmar, which diversified the rice paddy landscape. While this was unexpected, low levels of industrialization in agricultural activities in Myanmar, compared to Thailand or other places in Southeast Asia, can also explain these differences. High levels of agricultural intensity typically decrease bird species richness and diversity as was shown in northern Myanmar (Platt et al., 2021).

Proximity to coast and mangroves in Myeik may explain some locally specific differences in species diversity and composition in comparison towards Mandalay and Mawlamyine. The forest hill site in Myeik is an island while the other two forest sites are surrounded by agriculture and settlements with less sharp habitat edges. This fact could explain the relatively higher species diversity for Myeik forest habitat (Fig. 3). In the forest edge we found many bird species associated to water or mangroves, which we did not observe in the other two forest sites in the other cities. Distance to salt water (coastal sites) was also varying in some way, however low.

The relatively few species found in Mandalay and Mawlamyine forest sites may be related with the relatively small sample size, at least this might be the case for Mawlamyine. Although sample completeness was achieved for urban, suburban, and agriculture in all three cities, that was not the case for forest in Mawlamyine (Fig. 2). Alternatively, in Mandalay and Mawlamyine there is daily disturbance by humans in the forest site due to the religious activities. The human disturbance could trigger an anti-predator response by the birds (Stankowich & Blumstein, 2005), which could make them secretive thus difficult to record. In addition, very dry conditions associated with the survey seasons (at least for Mawlamyine) could also explain this result. Dry conditions may have driven the birds into more favorable areas. The only study addressing seasonality in Southeast Asia was from Singapore (Castelletta, Thiollay & Sodhi, 2005; Chong et al., 2012), all other studies had focused on a single year for evaluating the bird community. This can pose an issue, since bird communities can change in composition between seasons relatively quickly. However, with the data available from our study, there seems to be no temporal nor seasonal effect visible as Mandalay was sampled in a different season than Mawlamyine and Myeik, but this was not evident in the patterns seen (*e.g.*, Fig. 3).

Relative abundance was higher in urban sites in all three cities, as expected, while species compositions shifted from specialists in rural areas to more generalists along the rural to urban gradient, confirming our second hypothesis. Rock Pigeon was quite abundant in all three cities as also have been shown in any other urban city center throughout South and Southeast Asia as well as worldwide (Aronson et al., 2014). Besides Rock Pigeon, Common Myna and Eurasian Tree Sparrow are typically among the most frequently observed bird species close to the city centers and less abundant (or not observed) in other habitat types or in the surroundings of large cities of South and Southeast Asia (Chaiyarat et al., 2018; Chong et al., 2012). Similarly, in our study few species have many records and many species have single or a few records.

For birds within urban areas of Myanmar, only one study from Mandalay exists. We found overlapping similarities and our results largely support the findings of this previous study from Mandalay (Suarez-Rubio et al., 2016). In Mandalay the most frequent species, Rock Pigeon and House Sparrow, were equally frequent in 2015 (Suarez-Rubio et al., 2016) and in 2019, our current study. Differences in species diversity and composition might be driven by a seasonal difference of the two assessments (November 2015 *vs.* June 2019). This could be seen partially in migrant-species causing locally a seasonal shift in 2015, although the pattern along the urban to rural gradient persisted.

Similar to what was found in other large cities of South and Southeast Asia, such as Kolkata (Sengupta, Mondal & Basu, 2014), Singapore (Castelletta, Thiollay & Sodhi, 2005; Chong et al., 2012), and Bangkok (Chaiyarat et al., 2018), in Myanmar urban core habitats had more generalist species and were less diverse compared with peripheric habitat types such as forests. Generalists species (*e.g.*, Rock Pigeon) find human-associated food and nesting resources in urban core areas compared with the periphery. These species are replaced by more specialized species such as forest dwelling or piscivores towards the periphery. The shift in species composition from generalist species to specialized species along the urban to rural gradient may highlight the importance of green infrastructure in particular its vegetation structure and size for supporting bird diversity in urban and suburban areas (Beninde, Veith & Hochkirch, 2015; MacGregor-Fors et al., 2016).

From South Asia, several studies are available, but mainly from Kolkata (Sengupta, Mondal & Basu, 2014). In Kolkata a total of 48 bird species were recorded in 2,858 individuals but the species diversity (Shannon–Wiener) was independent of habitat or land use (Sengupta, Mondal & Basu, 2014). In Myanmar, the bird communities were clearly distinct between the rural and urban sites, and indeed we found congruency in relative abundance between Mandalay, Mawlamyine, and Myeik. Our models confirm general findings, that increasing amount of impervious surface has a negative effect on relative abundance of birds, whereas proportion of green has a positive effect. We did not find any indication of a similar pattern for the urban bird communities, neither in Myanmar nor in the neighboring countries of Southeast Asia.

We could partially confirm our third hypothesis, *i.e.,* functional groups varied along the urban to rural gradient and omnivores were dominant towards the urban core and decreased towards the periphery. However, for most functional groups, the higher relative abundance was encountered in medium land use intensity (*i.e.,* suburban and agriculture). This has not yet been shown by the other mentioned studies in South and Southeast Asia. But these studies as well as ours show that generalists—mostly omnivores—are positively associated with urban places. Urban bird communities are usually dominated by granivores, omnivores, and less by other groups in temperate areas (Beissinger & Osborne, 1982; Blair, 1996; Fernández-Juricic, 2001; Ortega-Alvarez & MacGregor-Fors, 2009). While we also found mainly omnivores, and to some extend granivores in urban downtown, insectivores were very common in our study supporting the findings that this groups is predominant in some tropical and subtropical urban areas (Aronson et al., 2014; Ortega-Álvarez & MacGregor-Fors, 2011). All other functional groups were favorably found in areas with medium-high urbanization—suburban and/or agriculture, suggesting that these areas are not as intensively used as elsewhere (Chace & Walsh, 2006).

Our results support previous studies in which higher abundance of few species are found in areas with high human population and housing density (Suarez-Rubio, Leimgruber & Renner, 2011; Suarez-Rubio et al., 2016). We show, however, that urbanization in Myanmar is altering species diversity, species abundance and species composition, of birds, and that generalists bird species are more frequent towards the urban core, while functionally more specialized groups increase towards the periphery. The latter aspects have not been shown for Southeast Asian cities so far.

## Supplemental Information

10.7717/peerj.16098/supp-1 Table S1 Count data form the three cities and four habitat types in 2019

10.7717/peerj.16098/supp-2 Figure S1 NMDS ordination for 3 cities in Myanmar Mandalay, Mawlamyine, and Myeik

10.7717/peerj.16098/supp-3 Figure S2 NMDS ordination for 3 cities in Myanmar, for of fucntional groups in rspect to species richness in (A) for Mandalay, (B) Mawlamyine, and (C) Myeik and for relative abundance in (D) Mandalay, (E) Mawlamyine, and (F) Myeik

## Additional Information and Declarations

### Data Availability

The following information was supplied regarding data availability: The raw data is available in the Supplemental File.
