# Supplementary figures and images for: Bird diversity along an urban to rural gradient in large tropical cities peaks in mid-level urbanization
Source: PeerJ. 2023 Oct 9;11:e16098. doi: 10.7717/peerj.16098 (PMC10569181; doi:10.7717/peerj.16098)

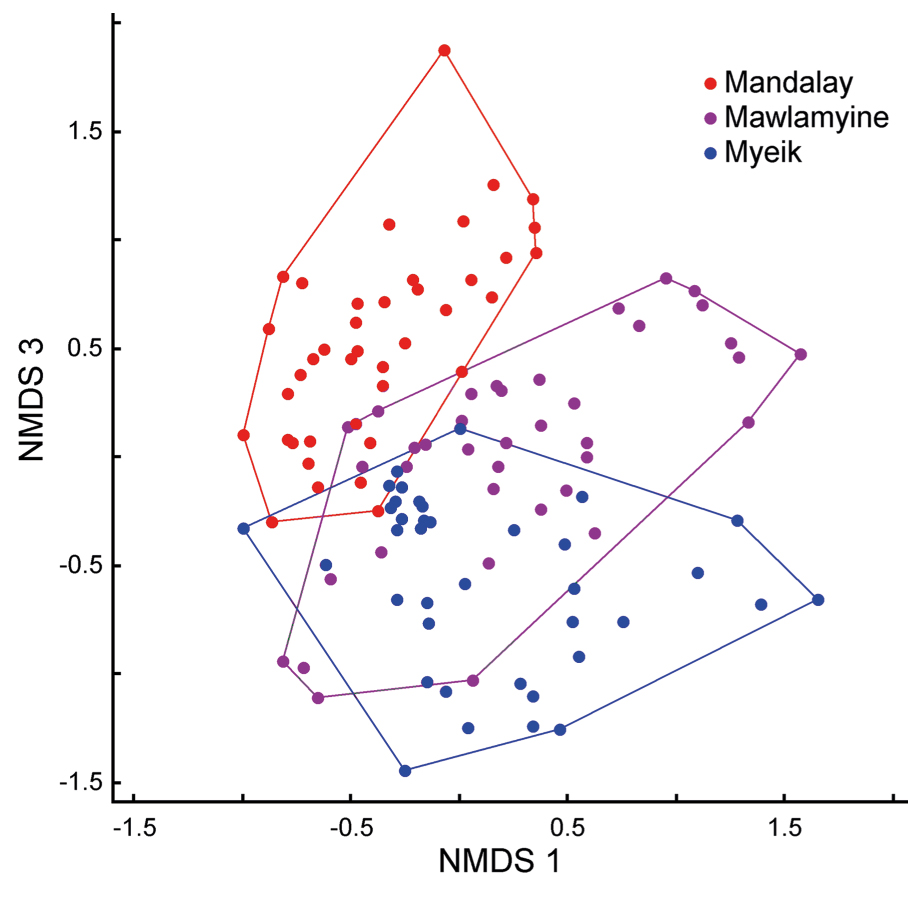

Supplement: Figure S1 [file peerj-11-16098-s002.jpg]

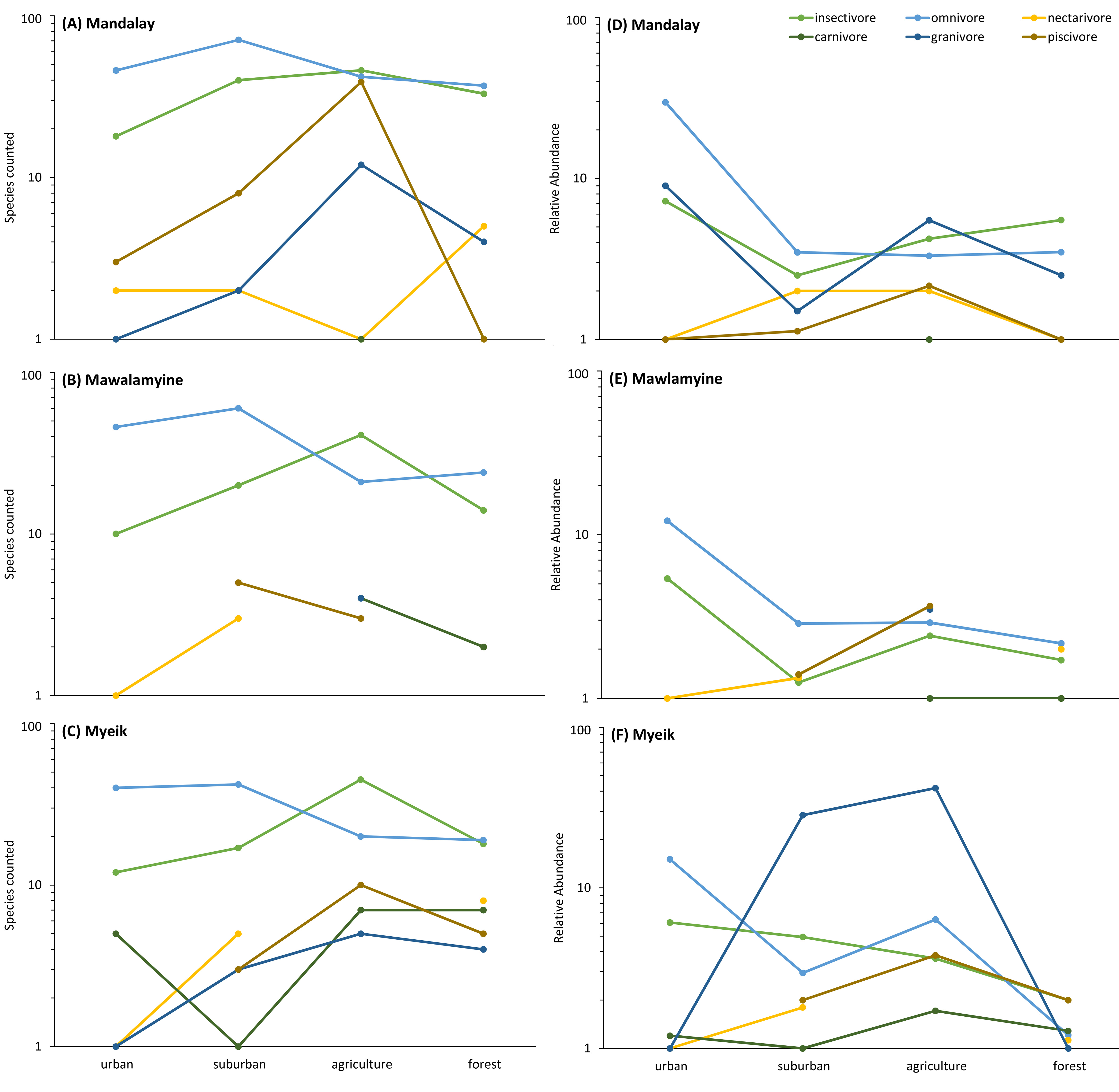

Supplement: Figure S2 [file peerj-11-16098-s003.jpg]
